# P42 From drips to decisions: an analysis of IVOS in an acute NHS district hospital, and the barriers preventing it

**DOI:** 10.1093/jacamr/dlaf118.049

**Published:** 2025-07-14

**Authors:** Alice Liu, Maria Girgis, Owen Harris, Rachel Pye

**Affiliations:** Gloucestershire Hospitals NHS Foundation Trust, UK; Gloucestershire Hospitals NHS Foundation Trust, UK; Gloucestershire Hospitals NHS Foundation Trust, UK; Gloucestershire Hospitals NHS Foundation Trust, UK

## Abstract

**Background:**

IV-to-oral-switch (IVOS) is a multidisciplinary approach in which patients who are treated with IV antibiotics are stepped down to oral antibiotics, providing they are afebrile, clinically improving, eating and drinking and not suffering with a deep-seated infection (ACED). The IVOS of antibiotics for inpatients is a fundamental decision in both upholding antimicrobial stewardship and expediting discharge; the switch also reduces operational and financial pressures on the trust. The latest NHS planning guidance brings an intensified financial demand on the NHS and for this reason, more than ever, the cost-saving opportunities within effective IVOS should not be overlooked.

**Objectives:**

To analyse the IV antibiotic prescribing against drug cost and highlight the cost saving opportunities if IVOS took place; and to identify the barriers preventing this in practice and make recommendations for improvement.

**Methods:**

The patient list on IV antibiotics over 24 h was extracted from Business Intelligence (BI) and data was analysed against the electronic patient record (EPR) using the modified version of the UKHSA Antimicrobial IV-to-oral (IVOS) decision aid tools^1^ as a snapshot study across the whole hospitals. Patients on IV antibiotics for prophylaxis, antifungal and antiviral treatment and patients in intensive or high-dependency units are excluded in the study.

**Results:**

Thirty-six out of 110 patients (33%) met the criteria^1^ for IVOS and the average length of IV antibiotic course was 5.7 days in total (range 2-21 days). Twenty-five patients (23%) were found to be able to switch to oral antibiotic(s) sooner than the audit day which meant that a total of 39 hospital-bed days could be saved if they were medically fit for discharge. Surgery in digestive tract used the highest volume of IV antibiotics which contributed to 14.5% of all IV antibiotic prescriptions (*n*=159), 53% of these patients were suitable for IVOS. The highest rate of IVOS was seen in acute medicine with 3 out of 5 patients have changed to oral antibiotics on the audit day. The most common reason for being unsuitable for IVOS was ‘one or more infection marker criteria not met’. The total expenditure of IV antibiotics was £5348.83 in which £1124.55 (21%) was spent on piperacillin-tazobactam; the most commonly used antibiotic in the study.

**Conclusions:**

The results highlight key opportunities to maximize IVOS efficiency, by directing resources toward high IV antibiotic prescribing specialities such as GI Surgery and regular review of broad-spectrum antibiotics such as piperacillin-tazobactam. IVOS should be advocated through educational and training to the doctors and nurses, regular antimicrobial stewardship ward round and advocacy of IVOS through hospital staff communications. The use of artificial Intelligence should be maximized to help ‘detect’ the patients on IVOS whom met the criteria for oral switch and alert the clinical team for prompt review which could help reducing the unnecessary use of IV antibiotic and promote hospital discharge.
